# Cruel Treatment of a Female Patient by Her Parents

**Published:** 1887-08-13

**Authors:** 


### CRUEL TREATMENT OF A FEMALE PATIENT BY HER PARENTS.

In reference to the question of our correspondent in last week's issue, "May a young lady be detained in an asylum against her parents' wishes?" it may prove of interest to many to republish the following particulars of a case which is given under the above heading in the report of the Lunacy Commissioners recently issued :—

"Early in March last we received a communication from the chairman of the police committee of a county of England, calling attention to the circumstances attending the then very recent admission to the county asylum of a lady sent thither by two justices, as a lunatic 'cruelly treated by those in charge of her.'

"The circumstances were these : The patient in question resided in the house of her father. The alleged cruel treatment consisted in keeping the lunatic fastened down in bed night after night by a jacket of peculiar construction, which almost entirely prevented any movement of the head, body, or limbs. This treatment had been resorted to for several months in 1879, and again, upon a recurrence of the attack, from February 1885 to February 1886, the apparatus not being removed on one occasion for six weeks successively. During this time no medical advice had been taken by the parents. The patient, it should be stated, was kept clean, was properly fed, and had, generally, a nurse attending her.

"The monstrous system of mechanical restraint above described seems to have been put into operation in the belief that it was necessary in order to prevent objectionable practices to which the patient was at certain times disposed ; yet at the asylum the medical superintendent found no occasion to resort to any mechanical restraint in her case.

"Although it seemed to us clear that Mr. and Mrs. —— had been guilty of treating their daughter in a way which amounted to cruelty and neglect, whatever the motives of their conduct may have been, we were in some doubt as to the advisability of taking criminal proceedings against them, feeling sure that the case of Reg. *v.* Rundle would be pressed against us. This case was in 1885, and in it the Court for Criminal Cases Reserved held that the words in Section 9 of the Act 16 and 17 Vict., c. 96, 'persons having the care and charge of a lunatic,' are to be construed so as not to include a husband (or apparently any other person) 'having the care or charge of the lunatic by reason solely of the domestic relation subsisting between them.'

"Upon full consideration, however, we determined to summon the parents for cruelty and neglect. The case was heard at the Petty Sessions in April last, when the defendants were convicted, and fined £20 each, with costs. The Justices, however, stated a case on the point of law for the consideration of the Queen's Bench Division.

"The case was not argued and decided until 10th March 1887, while this report was in preparation. On account, however, of the great importance of the matter, and in the interests of the insane, we think it well to anticipate our report for 1887 by stating that the conviction was affirmed, and that it is now clearly the law that the parents of a lunatic who resides with them under their care are persons 'having the care and charge' of a lunatic within the meaning of 16 and 17 Vict., c. 96, s. 9, and may be convicted under that section for ill-treating such lunatic."

